# Anti-factor H antibody and its role in atypical hemolytic uremic syndrome

**DOI:** 10.3389/fimmu.2022.931210

**Published:** 2022-08-23

**Authors:** Rupesh Raina, Guneive Mangat, Gordon Hong, Raghav Shah, Nikhil Nair, Brian Abboud, Sumedha Bagga, Sidharth Kumar Sethi

**Affiliations:** ^1^ Department of Nephrology, Akron Children’s Hospital, Akron, OH, United States; ^2^ Department of Nephrology, Akron Nephrology Associates/Cleveland Clinic Akron General Medical Center, Akron, OH, United States; ^3^ Department of Medicine, Northeast Ohio Medical University, Rootstown, OH, United States; ^4^ Department of Medicine, Ohio States University, Columbus, OH, United States; ^5^ Department of Medicine, Case Western Reserve University School of Medicine, Cleveland, OH, United States; ^6^ Questrom School of Business, Boston University, Boston, MA, United States; ^7^ Paediatric Nephrology & Paediatric Kidney Transplantation, Kidney and Urology Institute, Medanta, The Medicity Hospital, Gurgaon, India

**Keywords:** anti-factor H antibody, atypical hemolytic uremic syndrome, aHUS, pediatric, factor H

## Abstract

Atypical hemolytic uremic syndrome (aHUS) an important form of a thrombotic microangiopathy (TMA) that can frequently lead to acute kidney injury (AKI). An important subset of aHUS is the anti-factor H associated aHUS. This variant of aHUS can occur due to deletion of the complement factor H genes, *CFHR1* and *CFHR3*, along with the presence of anti-factor H antibodies. However, it is a point of interest to note that not all patients with anti-factor H associated aHUS have a *CFHR1/R3* deletion. Factor-H has a vital role in the regulation of the complement system, specifically the alternate pathway. Therefore, dysregulation of the complement system can lead to inflammatory or autoimmune diseases. Patients with this disease respond well to treatment with plasma exchange therapy along with Eculizumab and immunosuppressant therapy. Anti-factor H antibody associated aHUS has a certain genetic predilection therefore there is focus on further advancements in the diagnosis and management of this disease. In this article we discuss the baseline characteristics of patients with anti-factor H associated aHUS, their triggers, various treatment modalities and future perspectives.

## Introduction

Atypical hemolytic uremic syndrome (aHUS) is an important form of a thrombotic microangiopathy (TMA) with a chronic onset. It is an important etiology of acute kidney injury (AKI) and end-stage kidney disease (ESKD). Out of all typical hemolytic uremic syndrome (HUS) cases in children, 5-10% are recurrent cases of aHUS (1). Around 50% of aHUS cases arise from genetic mutations that encode regulatory proteins of the alternate complement pathway such as complement factor (*CFH*), complement factor I (*CFI*), membrane cofactor protein (*MCP*) as well as mutations in genes of complement component such as complement factor B (*CFB*) and *C3.* Factor H (FH) is one of these important regulatory proteins. Anti-FH (aFH) antibody associated aHUS is a unique subgroup of aHUS occurring at any age, but it is more prevalent in the pediatric population. These patients develop auto antibodies that bind to the C-terminus of FH thus impairing the interaction of FH with C3b and thereby causing dysregulation and overactivity of the complement pathway. This compromised interaction of FH and C3b is a significant step in the pathogenesis of this disease because it halts the amplification of the alternate pathway (2). These antibodies have an impairing effect on regulatory function of FH (2). It is imperative to diagnose aFH antibody associated aHUS quickly and to provide timely treatment to patients. In this article, we aim to discuss patient baseline characteristics, prevalence of aFH antibodies, treatment modalities and future perspectives.

## Importance of anti-factor H antibodies

**Figure 1:**
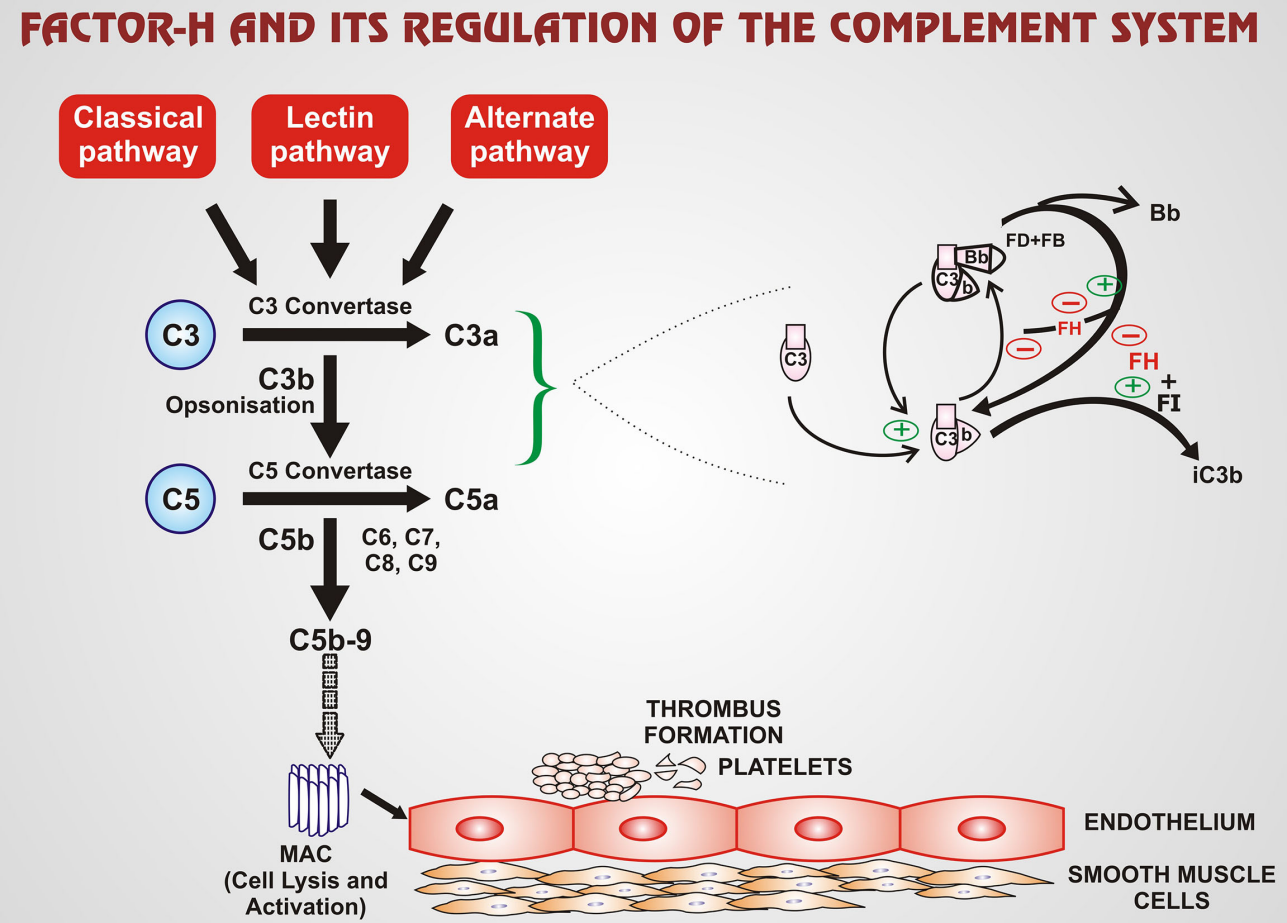
FH is an important regulatory protein of the complement pathway, with its effect mainly on the alternate pathway of the complement system. FH regulates the complement system *via* two mechanisms: DAA and CA. DAA enables FH to displace Bb fragment of FB off C3 convertase. Thus, the accelerated irreversible decay of C3bBb into C3b and Bb takes place. In the CA mechanism, FH has the role of a facilitator in the FI-mediated cleavage of C3b to iC3b, which is an inactivated form of C3b. The green ‘+’ arrows exhibit stimulatory or enhanced action whereas red ‘-’ arrows exhibit inhibitory action. Thus, over-amplification of the complement pathway is regulated by FH. The C5b-9 is one of the terminal components of the MAC which mediates the cell lysis and activation completing the complement cascade. The broken arrow implies that MAC enhances the process of thrombus formation from platelets which consists of a series of steps. (FH-factor-H; FI-factor-I; FB-factor-B; FD-factor-D; MAC-membrane attack complex; DAA-decay accelerating activity; CA-co-factor activity; iC3b-inactivated C3b).

aFH antibodies present in aHUS in about ~20% of patients. Patients usually have some other genetic abnormalities as well. 50% of the aHUS cases arise due to genetic mutations. The complement system is a regulatory system of the innate immune system that clears pathogens, immune complexes, and apoptotic cells. It is important to highlight and analyze the biological features, and clinical manifestations of aFH antibodies, and the treatment guidelines to prevent relapse of disease and renal failure. FH is a significant fluid-phase and cell surface regulatory protein that protects from uncontrolled complement activation by serving as a cofactor in cleavage of C3b and accelerating decay of C3 convertase (3). FH carries out this complement regulation through two different mechanisms- decay accelerating activity (DAA) and co-factor activity (CA) (4). DAA enables FH to aid in the displacement of Bb fragment of factor B (FB) off C3 convertase thus accelerating the irreversible decay of C3bBb into C3b and Bb. In the CA mechanism, FH assumes the role of a facilitator in the factor-I (FI)- mediated cleavage of C3b to iC3b, which is an inactivated form of C3b (4). This complement regulation carried out by FH is also portrayed in **Figure 1** given below. The FH family is completed by a total of six FH- related proteins which include FHR 1-3, FHR 4A, FHR 4B and FHR 5. These proteins are encoded by genes present as a cluster located on 3’ of FH. The genes are *CFHR1*, *CFHR2, CFHR3, CFHR4*, and *CFH5*. Genomic duplication of these genes lead to undesirable effect on FH thereby impairing its various functions (3). FH is made up of 20 short consensus repeats (SCRs) with two binding sites for C3b, the principal regulatory molecule of the complement pathway. The first binding site is in the SCR 1-4 N-terminal which controls complement pathway amplification. The second binding site is in the SCR 19-20 of the C-terminal domain and has a pivotal role in protecting endothelial cells (3). Most often the aFH autoantibodies bind to the SCR 19-20 region thereby compromising it (3). Earlier research found the aFH antibody to bind to the C-terminal domain, blocking this site from interacting with FH. The N-terminal domain has also been demonstrated to be impaired by aFH antibodies, however the C-terminal is more frequently implicated. The interaction of FH and C-terminus is critical for FH to be docked into C3b covered cells or surfaces and for FH to regulate the complement system. Therefore, the aFH antibodies can contribute to impairing function of FH and thereby causing misguided complement activation (5). Since, proper binding between FH and C3b is inhibited, this dysregulation and overactivation of the complement system causes damage to host cells such as blood cells or endothelial cells (6, 7). Immunosuppression may be a successful treatment option in patients with detectable aFH antibodies. aFH antibodies may be associated by a homozygous deletion in the *CFHR1* and *CFHR3* gene, with *CFHR1* gene being more importantly linked, resulting in a loss of function mutation and inducing an impairment in the function of FH. While some FH antibodies are linked with *CFHR1* gene deletion, recent studies have shown that not all patients with *CFHR1* deletion will display aFH antibodies. It can also be stated that the presence of aFH antibodies does not imply a *CFHR1* deletion. Therefore, this gene deletion alone is not sufficient enough to give rise to the disease process. Recent studies have speculated that mutations in complement regulators/activators may also be associated with aFH-antibodies (8). In a study by Moore et al. mutations specifically in FH, C3, membrane cofactor protein (MCP), and complement factor I (CFI) have been shown to be associated with FH autoantibodies aHUS. Mutations were seen in five of 13 patients with FH autoantibodies. One patient was observed with a FH mutation, one with a CFI mutation, and two other patients with C3 variants without deletions in *CFHR1* or *CFHR3* (9). This study correlates similarly to the cohorts of Hofer et al. with three patients were positive for FH antibodies without deletion in *CFHR1* (10). In total, nearly 10% of the patients with aFH antibodies do not show a deletion of gene *CFHR1*. aFH antibody associated aHUS is a relatively newer subset of aHUS. Therefore, it is crucial to analyze aFH associated aHUS clinical outcomes and patient characteristics to highlight functional implications which can help predict relapse (6).

## Prevalence/incidence of anti-FH-associated aHUS

aHUS is a significant cause of AKI in the pediatric population. Abnormalities in the complement and coagulation systems are associated with aHUS. According to a study by Bagga et al. in 2019, aFH antibodies were detected in approximately 50% of pediatric patients diagnosed with aHUS, typically in the 5-15 year old age group (11). This subset of aHUS is prevalent in 5-25% of patients with aHUS in European and North American cohorts, and ~50% in India (12). This study represents one of the largest cohorts for this population setting with homogenous clinical features given. While long-term outcomes were not followed for all the patients, thus limiting result generalizability. They do give a good stepping stone for diagnostic criterion and indications. Furthermore, data from the global aHUS registry reports the presence of aFH antibodies is 24% in all children with aHUS and 19% in adults diagnosed with aHUS (11). The deletion of *CFHR1* gene can be seen in 5-10% of healthy individuals in the world (13). As this complement abnormality is common in aHUS patients, it is imperative to analyze its biological features.

## Patient characteristics and triggers

aHUS can be triggered by an infection, resulting in a prodrome of a respiratory or diarrheal illness before disease onset. However, the aFH subset of aHUS does not have a diarrheal prodrome differentiating it from the other types of aHUS. Clinically, patients may suffer from an array of symptoms including anuria, oliguria, hematuria, elevated transaminases, low hemoglobin (<10 g/dl), low platelet count (<150 x 10^3^/mm^3^), neurological disturbances, and increased serum creatinine. Pediatric patients often show symptoms like pallor, lethargy, refusal to feed, vomiting and drowsiness most commonly. In contrast adult patients often report symptoms of fatigue and general distress (14).

More recently, Khandelwal et al. has shown infection with COVID-19 to be a potential trigger in the presentation of aHUS (13). During the second wave of the pandemic in India, five patients ages 4-13 years were found to have aFH antibody associated aHUS coinciding with a diagnosis of COVID-19. All patients had detectable aFH antibodies with high titers (mean 2,300AU/ml). Three of these patients presented as a relapse of aHUS after a 2–6-year period of disease quiescence following an initial episode. Three of the five patients also showed a *CFHR1* gene deletion on genetic testing. This suggests aFH associated aHUS patients were at an increased risk of suffering a relapse or recurrence after contracting COVID-19 infection, despite a long dormant course (13). While showing promising results, this paper is limited by its small patient population.

In 2019, Puraswani and Bagga et al. conducted a study highlighting the clinical features of 781 patients with aHUS under the age of 18 (11). Of these patients, 436 were observed to have aFH antibody associated aHUS. This study showcases the most prevalent features of patients at presentation: 131 patients (30.0%) had anuria, 45 (10.3%) and 29 (6.7%) had respiratory and gastrointestinal illness respectively. 162 (37.2%) patients exhibited elevated transaminases. Stage 2 hypertension was observed in 238 (54.6%) of the patients. The mean values for hemoglobin, platelet count and serum creatinine were observed as 5.3 ± 1.3 g/dl, 59.9 ± 39.1 x 10^3^/mm^3^ and 5.56 ± 2.98 mg/dl respectively. aFH antibody titers in these patients were 10,633.2 ± 998.5 AU/ml (11). This study does suffer from heterogeneity in regard to patient population as management was done at the discretion of the physician.

**Table 1** depicts baseline characteristics seen in patients with aFH antibody associated aHUS along with their incidence. 18 studies with a total of 3130 patients have been represented in this table, each showcasing the number of patients with positive titers for aFH and the most prevalent clinical features. Anuria was observed to be present in 196 patients out of the total patients making it the most common feature. **Table 2** summarizes aFH antibodies in 16 studies with 95% confidence intervals.

**Table 1:** aHUS patients with anti-FH antibodies across different studies.

Study	aHUS patients w/Anti-factor H antibody (n)	Anuria	Elevated transaminases	Hypertension	Hemoglobin (g/dl)	High creatinine (mg/dl)	Prodromal illness (vomiting, respiratory illness, fever)	Platelet count (x 10^3^/mm^3^)
Puraswani M et al., 2019 (11)	436	131 (30%)	162 (37.2%)	238 (30.4%)	5.3 ± 1.3	5.56 ± 2.98	312 (39.9%)	59.9 ± 39.1
Valoti E et al., 2019 (15)	30	–	–	–	7.3 ± 1.4	5.7 ± 3.3	23 (73.3%)	61.923 ± 41.218
Lee et al., 2015 (16)	15	27 (53%	5 (10%)	24 (47%)	7.6 ± 3.4	2.9 (0.4~15.1)	23 (45%)	69 (9~129)
Fremeaux-Bacchi et al., 2013 (17)	14	–	5 (6%)	–	–	–	83 (38.7%)	–
Hofer et al., 2013 (10)	30	9/18 50%	–	10/17 (59%)	5.8 ± 1.5	5.5 ± 3.2	13/15 (87%)	30.0 ± 13
Guo W et al., 2019 (5)	36	28 (78%)	17 (47%)	29 (81%)	6.77 ± 1.83	–	30 (83%)	38.5 ± 23.2
Brocklebank et al., 2017 (18)	17	10 (5.88%)	2 (11.76%)	9 (52.9%)	6.6 (4-9.1)	–	16 (94.1%)	52 (9-134)
Hofer et al., 2013 (10)	19	–	–	13/18 (72%)	5.7 ± 1.1	4.4 ± 3.1	17/19 (89.4%)	29 ± 12
Tiewsoh et al., 2021 (19)	15	–	–	14/15 (93.3%)	5.8 ± 1.0	4.99 ± 2.49	–	58 ± 38.7
Shawky et al., 2021 (2)	12	12/12 (100%)	–	2/12 (16.7%)	7 ± 0.7	6.2 ± 4.6	10/12 (83.3%)	79.1 ± 34
Song et al., 2017 (20)	33	–	–	27/33 (81.8%)	6.77 ± 1.83	3.63 ± 2.72	28/33 (84.8%)	38.5 ± 23.2

**Table 2:** A summary of anti-FH antibodies in aHUS patients over 16 studies with 95% confidence intervals.

Study	Event Size/Sample Size (presence of Anti-FH Ab)	Estimated % (95% CI)
Puraswani M et al., 2019 (11)	436/781	55.83 [0.5273, 0.05893]
Valoti E et al., 2019 (15)	30/305	9.83 [0.0000, 0.1993]
Bernabéu-Herrero et al., 2016 (21)	14/367	3.81 [0.0000, 0.1472]
Lee et al., 2015 (16)	15/51	29.41 [0.0801, 0.5081]
Fremeaux-Bacchi et al., 2011 (17)	14/214	6.54 [0.0000, 0.2034]
Hofer et al., 2013 (10)	30/116	25.86 [0.1236, 0.3936]
Noris et al., 2010 (22)	8/273	2.93 [0.0000, 0.1683]
Durey M et al., 2009 (23)	14/177	7.91 ([0.0000, 0.2291]
Moore I et al., 2009 (9)	13/142	9.15 ([0.0215, 0.1615]
Józsi M et al., 2008 (24)	16/147	10.88 [0.0000, 0.3850]
Durey M et al., 2005 (25)	3/48	6.25 [0, 0.651]
Jozsi M et al., 2007 (26)	5/51	9.80 [0, 0.453]
Gurjar et al., 2018 (27)	98/164	59.76 [0.536, 0.659]
Brocklebank et al., 2017 (18)	17/175	9.71 [0, 0.242]
Shawky et al., 2021 (2)	12/26	46.14 [0.224, 0.698]
Song et al., 2017 (20)	33/156	21.15 [0.087, 0.336]
Total Random Effects	758/3160	23.9 [0.213, 0.266]

## Clinical outcomes/mortality of anti-factor H antibody aHUS

Bagga et al. found that aHUS patients had clinical symptoms such as hypertension, chronic kidney disease (CKD) and mortality was discussed (11). At a three-month follow-up post treatment, 152 (42.7%) patients were noted to have stage-2 hypertension, with 64 (18%) of them developing CKD stage 2-3 and 81 (22.8%) experiencing adverse outcome namely CKD stage 4-5 and death. This study also compared the titers of aFH antibodies from the onset of disease and through various stages at subsequent follow-ups. aFH antibody titer at onset of disease in 44 subjects was a mean of 5,000 AU/ml (ranging from 2,121-163,829 AU/ml.) The titers at follow-up at three months (n=42), six months (n=47) and 12 months (n-=23) were 409 (254-861), 277 (154-893.6) and 408 (262-691.8) respectively. The number of days from onset to hematological remission on average was 27 (17-41) days (11). Titers were not drawn from all of the patients within the study and need to be examined on the total cohort to determine their full significance. Similarly, a cohort study of 38 children and seven adults with aFH antibody associated aHUS done was by Dragon-Durey et al. In long term follow-ups (39 months on average), patients showed spikes in renal complications: end-stage renal disease in 27% of patients and mortality of 9.1% (23). Khandelwal et al. conducted study including 45 patients with aFH antibody associated aHUS who were followed-up for >12 months. These patients were treated with plasma exchange therapy (PEX) and exhibited a decline in antibody titers from a mean of 3215.5 AU/dl to 414.6 AU/dl. The serial antibody examinations revealed no difference in decline of titers following immunosuppressive treatment with cyclophosphamide or rituximab. Both drugs had comparable outcomes (28). Comparisons need to be done with patients not exhibiting aFH antibodies to compare their effect on clinical outcomes.

## Treatment modalities

A patient suspected of having aFH antibody associated aHUS, should be transferred to a specialized center with intensive care and dialysis provisions in case of complications. Over the years, many therapies have been used to manage aHUS such as plasma exchange therapy, eculizumab, immunosuppressants like prednisone, cyclophosphamide, rituximab and eventually a renal transplant. According to a study conducted by Bagga and Khandelwal et al. in 2019, plasma exchange therapy (PEX) along with immunosuppressants is the recommended treatment for aFH antibody associated aHUS (13). This study, however, did not show a significant change in renal outcomes through PEX use *vs*. without PEX is done using fresh frozen plasma as a replacement fluid. The use of plasma infusions alone is not recommended by these guidelines. In this disease, the aim is to decrease antibody titers. PEX helps to achieve up to 80% reduction in the antibody titers in an average 5-7 sessions (28). The American Society for Apheresis recognizes aFH antibody aHUS as a level I condition and thus recommends PEX as the primary treatment option (29). Along with decreasing antibody titers, PEX also replenishes the complement factors like FH and FI and removes the aFH antibodies from the circulation (30). However, treatment duration is unknown. Plasma therapy poses some challenges in children due to its high morbidity especially in very young children and those with a low-body weight. Catheter infections (50%) and catheter-related thrombosis (19%) were notable adverse effects seen in children in a study by Wijnsma in 2019 (31). Due to these adverse effects, it is vital to begin immunosuppressant therapy right after PEX.

Eculizumab is a monoclonal antibody (IgG) which binds to C5 complement protein and thereby prevents its cleavage further into C5a and C5b. By preventing the formation of C5b, eculizumab blocks the formation of the membrane attack complex (MAC) (32). Eculizumab is preferred in patients who possess the FH mutations, and it further reduces the likelihood of recurrences (33). Eculizumab has also been recommended to be a safe intervention in the pediatric population for the treatment of aFH aHUS by Raina et al. (34) A study was conducted by Greenbaum et al. tested efficacy of eculizumab in 22 pediatric patients diagnosed with aHUS (35). These patients had a 26 week period follow up where. 18 patients achieved normalization of the hematologic laboratory parameters and 16 patients saw an improvement in their creatinine levels at the end of the follow up period.

Immunosuppressants like prednisone, cyclophosphamide, azathioprine, mycophenolate mofetil, and rituximab inhibit the formation of aFH antibodies (12). These immunosuppressants are used as maintenance therapy for aFH antibody associated aHUS. Antibody titers require close monitoring for 3-6 months due to risk of relapse following minor infections. Another reason for relapse of aHUS in patients is the cessation of plasma exchange therapy. According to a study done by Loirat et al. in 2011, there is a steep rise in the titers of aFH antibodies soon after plasma therapy cessation (32). The use of immunosuppressants lowers the risk of such an event (12). Immunosuppressive therapy is used to maintain remission in aFH antibody associated aHUS patients and reduce replace rate. However, there is not any current standardized duration for immunosuppressive therapy (32). In a study conducted by Dragon-Durey et al., 38 pediatric patents and seven adult patients with aFH associated aHUS were included. Out of these patients, five pediatric patients received immunosuppressive therapy. One child out of them relapsed at 2- and 7-months post onset of disease once plasma therapy was discontinued. This patient was treated with cyclophosphamide which allowed for a successful cessation of PEX and did not exhibit any relapses (23). In patients who undergo renal transplantation after aHUS, immunosuppressive therapy holds an important role in preventing graft rejection as well.

Dragon-Durey et al. conducted a study in 2010 to compare various treatment modalities with disease progression (23). Patients were treated with conservative treatment(n=6), plasma infusion alone (n=6), PEX therapy (n=15) or PEX plus immunosuppression (n=3). On follow up (mean 48 months), it was observed that all three patients who were treated with both PEX and immunosuppressive therapy did not relapse. In contrast, the patients treated with conservative therapy (2 of 6, 33%), plasma infusion (5 of 6, 83.3%) or PEX (6 of 15, 40%) alone exhibited relapse. This suggests that the most efficient therapy for aFH antibody associated aHUS is PEX therapy combined with immunosuppressant therapy (23).

In a study by Puraswani and Bagga et al., out of the 436 aFH antibody aHUS patients, plasma exchange therapy was performed for 72.7% patients for a period of 14 days. This was followed by immunosuppression therapy with prednisone and cyclophosphamide or rituximab. During the six-year period patients showed improved hematological status and achieved remission with this therapy (11). As per the Indian guidelines in the paper by Bagga et al. in 2019, once PEX is started, induction therapy with oral steroids and IV cyclophosphamide (preferred) or IV rituximab is begun. Once hematological remission is achieved (decreasing antibody titers), PEX can be tapered down. The maintenance phase consists of immunosuppressant therapy with prednisolone for one year along with mycophenolate mofetil. Follow-up of antibody titers every 3-6 months helps monitor patients for relapse (12). While eculizumab is a first line treatment for aFH related aHUS in many countries, study done by Bagga et al. in 2019 suggest that it is indicated when patients exhibit a lack of remission despite multiple PEX sessions, life-threatening seizures, cardiac emergencies, complications with PEX or inherited flaws in the complement system (12). Another study by Noone et al. performed in patients with aFH antibodies showed that eculizumab added to treatment regimen decreased antibody titers (36). In one patient, the antibody titers decreased from 129 U/ml to 111 U/ml after eculizumab infusion. This drug was able to maintain a disease-free status in this patient previously dependent on PEX. In another patient, eculizumab was used after the patient suffered from an allergic adverse effect due to PEX (36). Loirat et al. state that eculizumab can be considered as an additional therapy in aFH aHUS patients as it provides a better renal recovery. It can also be given in cases of severe extra-renal manifestations such as serious cardiac or brain injury (37). Having discussed the various treatment modalities available and effective in this disease, there is still a lack of a centered and a well-established treatment protocol. There is a requirement for a standardized international system to follow in the treatment of this disease in acute stages as well as in the maintaining therapy to prevent recurrences.

## Kidney transplant in anti-FH aHUS

aFH associated aHUS has a relapsing course. The risk of developing end-stage renal disease and recurrence after kidney transplant is 30-40% (23). Kidney transplant in such patients is challenging due to high rates of recurrence. A study showing successful transplants was done by Khandelwal et al. to report the outcomes of those transplants in four patients with aFH antibody aHUS (38). Two patients underwent a cadaveric transplant whereas the remaining two patients underwent a living-related transplant. All patients had a history of relapses prior to transplantation and had undergone dialysis as well. The aFH titers in these patients prior to transplantation were 505 AU/ml, 1667 AU/ml, 2145 AU/ml and 294 AU/ml respectively. The values of these titers after PEX were not applicable in first patient, 589 au/ML, 1149 AU/ml, 152 AU/ml respectively in the other three patients. After transplant, there was further decline in titer values- 231 AU/ml, 181 AU/ml, 972 AU/ml, 126 AU/ml respectively. Three patients were on a maintenance therapy with prednisolone, however all patients had successful transplants with no relapse reported in any case. This leads us to the conclusion that kidney transplant might be an intriguing field of study and as a method for reducing the effects of this disease in patients (38).

## Future perspectives and scope

Given the strong predilection of aHUS with genetic abnormalities, genetics represents a vital field of study in this disease. There has been extensive work ongoing to study aHUS for over two decades. However there still is a paucity of methods to diagnose this disease more rapidly, hence, more advanced diagnostic modalities are required. In the pediatric population, aHUS often results in severe kidney damage therefore newer techniques must focus on quicker diagnosis to prevent AKI. PEX therapy in combination with immunosuppressant therapy has shown to be an effective management of aFH antibody associated aHUS. Additionally, there is a need for well-defined protocols regarding the role of anti-complement therapies like Eculizumab in this subset of patients versus immunosuppressive therapy. Eculizumab is not very readily available in many regions of the world for instance countries like India which has the greatest number of studies pertaining to PEX/plasmapheresis. In these instances, PEX/plasmapheresis along with immunosuppression would offer a hopeful alternative. In some cases plasmapheresis is unavailable, IVIG could be offered as an alternative, however there is a paucity of data and evidence to support this proposition. FH mediates numerous actions for the protection of our cells therefore it is imperative to expand the range of the research endeavors being carried out in the field. There is extensive research being undertaken in terms of genetic studies, gene editing, and biosimilar drugs. The current research studies should focus on quicker and more readily available diagnostic modalities for aHF associated aHUS. This will ensure timely management and rehabilitation of patients.

## Author contributions

GM and RS did initial literature search. RR laid the framework of the article. RR and SKS assisted in manuscript review. NN helped medically edit the manuscript and assisted with tables. GH and BA helped in critically reviewing the manuscript. SB and SS helped ensure final draft was suitable for submission. All authors contributed to the article and approved the submitted version.

## Conflict of interest

The authors declare that the research was conducted in the absence of any commercial or financial relationships that could be construed as a potential conflict of interest.

The handling editor C-YY declared a shared parent affiliation with the author RS at the time of review.

## Publisher’s note

All claims expressed in this article are solely those of the authors and do not necessarily represent those of their affiliated organizations, or those of the publisher, the editors and the reviewers. Any product that may be evaluated in this article, or claim that may be made by its manufacturer, is not guaranteed or endorsed by the publisher.
